# Dentists’ knowledge and practices in supportive care: a French national survey

**DOI:** 10.1007/s00520-026-10392-9

**Published:** 2026-02-23

**Authors:** Caroline de Bataille, Colas Prunier, Manon Saucourt, Joel B. Epstein, Emmanuelle Vigarios, Delphine Maret

**Affiliations:** 1 Department of Oral Medicine, Supportive Care Department, Oncopole Claudius Regaud, Institut Universitaire du Cancer Toulouse- Oncopole, Toulouse, France; 2 Department of Dental Surgery, Université de Toulouse, Toulouse, France; 3 City of Hope Comprehensive Cancer Center, and Cedars-Sinai Health System, Duarte, Los Angeles, CA USA; 4 Department of Dental Surgery, Laboratoire CAGT, Université de Toulouse, Centre Hospitalier Universitaire, Institut Universitaire du Cancer Toulouse- Oncopole Toulouse, 3 Chemin Des Maraîchers, 31400 Toulouse, France

**Keywords:** Supportive care, Dental care, Cancer, Oral complications, Professional education

## Abstract

**Purpose:**

Oral supportive care is essential in comprehensive cancer management, particularly for patients with head and neck cancer and those with hematologic cancers, who experience significant oral complications. While supportive care has been extensively explored in medical oncology, dentists’ knowledge and practices regarding oral supportive care for cancer patients remain understudied. This study aimed to assess French dentists’ knowledge, practices, and training needs regarding supportive care for cancer patients.

**Methods:**

A cross-sectional survey was distributed electronically to dental surgeons across France through Departmental Councils of the Order of Dental Surgeons. The questionnaire investigated practitioners’ knowledge of cancer patient management, care modalities, encountered difficulties, knowledge of supportive care, and training needs.

**Results:**

Among 165 respondents, 96% reported treating cancer patients, with head and neck cancers being significantly over-represented compared to general population incidence. Among respondents, 89% expressed concern about supportive care in oncology, while 73% rated their knowledge in this area below 5/10, and 27% were unaware of risks associated with cancer therapies. Head and neck cancers (54%) and hematological malignancies (15%) presented the greatest management challenges. The most common difficulties included performing invasive procedures (46.7%), writing prescriptions (45.8%), and completing medical questionnaires (40.2%). Only 29% of dentists reported having knowledge of supportive care in oncology, with 89% expressing a need for further training.

**Conclusion:**

French dentists demonstrate concern about their role in cancer patient care, yet face significant knowledge gaps and training deficiencies. Enhanced educational programs, improved interprofessional communication, and clear clinical guidelines specifically designed for dental professionals are needed to optimize supportive care delivery for cancer patients.

**Supplementary Information:**

The online version contains supplementary material available at 10.1007/s00520-026-10392-9.

## Introduction

Approximately 20 million new cancer cases are diagnosed worldwide each year [1]. Upon diagnosis, patients begin a care journey involving various treatments such as surgery, chemotherapy, radiotherapy, targeted therapies, and immunotherapy which are often associated with significant toxicities that persist over time. These toxicities substantially impact patients’ quality of life on both physical and psychological levels and may be underestimated [2]. The diverse toxicities associated with cancer treatments necessitate a multidisciplinary approach. In this context, supportive care has emerged as a critical component, involving various medical and paramedical professionals including physicians, nurses, pharmacists, psychologists, physiotherapists, dieticians, and others [3, 4].

Among the toxicities of anticancer treatments, many have direct or indirect consequences on the oral cavity and upon dental treatment [5]. Oral complications such as mucositis, xerostomia, taste loss, oral pain are common side effects of head and neck cancer treatments that can significantly impair oral function, nutrition, and speech, leading to a decline in quality of life [6]. More severe but less frequent complications, such as osteoradionecrosis, can also occur and further compromise patient outcomes. Consequently, dentists are called upon to intervene as early as possible to prevent or treat these complications [7].

Despite growing recognition of the importance of supportive care in dentistry, several limitations hinder its widespread implementation. These limitations include: (1) dentist-related factors such as insufficient knowledge of cancer therapy-related oral toxicities; (2) medical team-related factors including inadequate communication with dental professionals; (3) patient-related factors such as limited awareness of oral care importance; and (4) health system-related factors including limited access to specialized dental care and inadequate coordination between oncology and dental services [8, 9].

This study aimed to collect information on French dentists’ knowledge of cancer patient management, care modalities provided in dental offices, difficulties encountered with these patients, adaptations made compared to patients without cancer, and their knowledge and involvement in supportive care (SC).

## Materials and methods

### Questionnaire design

The questionnaire presented is a self-administered survey tool intended for dental surgeons. The questionnaire was validated by a panel of dental surgeons through pre-testing with 10 professionals to ensure question clarity. It is structured in multiple sections designed to collect information on their training, their management of patients with current or past cancer diagnoses, their perceived difficulties, their knowledge of supportive oncology care, and their personal assessment of their knowledge and training opportunities (online supplementary material). The questionnaire primarily employs closed-ended questions with predefined response options (single or multiple choice), as well as numerical rating scales (from 0 to 10) to measure difficulty, knowledge, and evaluation of training. Several open-ended questions also allow participants to elaborate on their responses. The language of the questionnaire is French.

The questionnaire was designed with four main sections:

- The first part collected demographic data and information about the respondents’ training and practice (6 questions).
- The second part analyzed how respondents manage cancer patients, including cancer locations and treatments encountered, care adaptations, and interdisciplinary communication (12 questions).
- The third section highlighted difficulties encountered by practitioners when treating cancer patients (5 questions).
- The final part assessed knowledge of supportive care, the dentist’s role in supportive care, and practitioners’ self-assessment of their knowledge and training needs in this area (8 questions).

The questionnaire was developed based on literature review and validated by a panel of dental surgeons through pre-testing with 10 professionals to ensure question clarity.”

### Questionnaire distribution

The questionnaire was distributed exclusively digitally via Google Forms® at the end of January 2021. A convenience sample of 500 dentists was selected from the mailing lists of the Departmental Council of the Order of Dental Surgeons of the Occitanie region. The Departmental Council of the Order of Dental Surgeons is the professional regulatory body that represents all licensed dentists in each French department. The Occitanie region was chosen as the study area because of its representative geographical diversity, including major urban centers (Toulouse, Montpellier) and rural areas, as well as the presence of leading cancer centers. The selection was made systematically from professional registration lists to ensure representativeness of different types of practices (urban/rural, generalist/specialist).

### Ethics declaration

This study was conducted in accordance with the ethical principles of the Declaration of Helsinki. All participants provided informed consent prior to participation in the survey. Data collection and processing were conducted in compliance with the General Data Protection Regulation (GDPR).

### Consent to participate

All participants provided informed consent electronically before completing the survey. The introductory page of the questionnaire explained the study purpose, the voluntary nature of participation, data confidentiality measures, and that completion of the questionnaire constituted consent to participate in the research. Participants were informed that their responses would be anonymized and used for research purposes only.

#### Data analysis

For open-ended questions, a posteriori categorization was performed to group similar responses for statistical analysis. Descriptive statistics were calculated for all variables.

## Results

### Respondent characteristics

A total of 165 dental surgeons responded to the questionnaire, out of 500 distributed, yielding a response rate of 33% (165/500). An average age of 48.3 years (SD ± 12.7), with 58% having practiced for more than 15 years. Sixty-two percent practice in urban areas, while 38% are in rural or semi-rural areas. The majority (89%) are general practitioners, with 11% having specialized training.

### Management of cancer patients

**Fig. 1:**
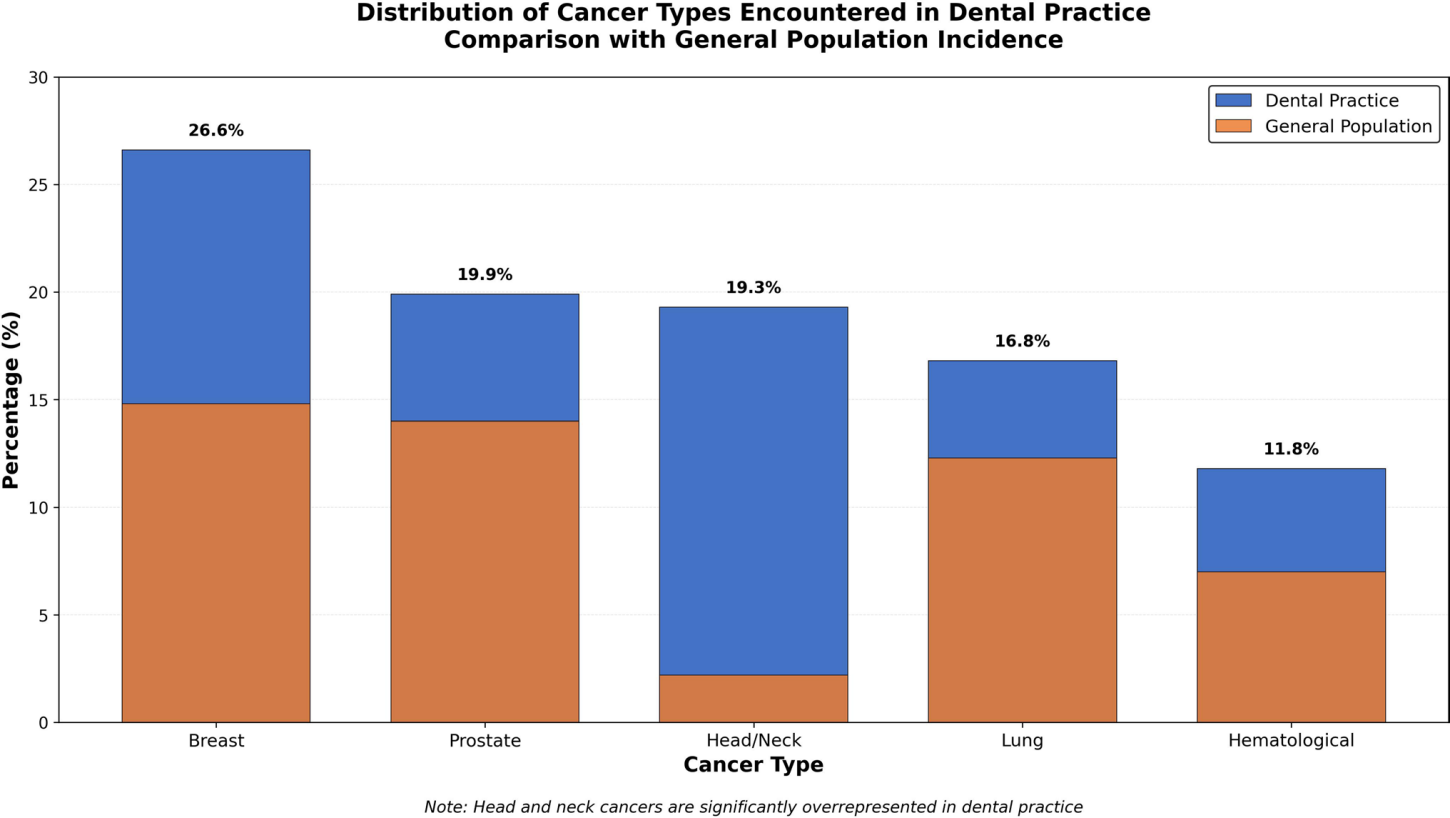
Distribution of cancer types encountered in dental practice. The figure shows the percentage distribution of different cancer types encountered by dentists, highlighting the overrepresentation of head and neck cancers compared to general population incidence [10]

Nearly all surveyed practitioners (96%) reported having patients with cancer in their practice. The most common cancer sites were breast (26.64%), prostate (19.88%), head and neck (19.31%), lung (16.80%), and hematological malignancies (11.78%) (Fig. 1).

The most frequently reported cancer treatments encountered in practice were chemotherapy (95%), radiotherapy (91%), and surgery (82%). Hormone therapy (54%), immunotherapy (43%), and targeted therapies (25%) were also commonly mentioned.

A concerning 27% of surveyed practitioners reported being unaware of risks associated with different therapies, and 58% stated they were unfamiliar with treatment-induced toxicities. Despite these knowledge gaps, 85% reported modifying their management approach for cancer patients and taking additional precautions. Sixty percent of practitioners contacted dental professionals at oncology centers. Among those who did, one-third sought information about patient treatment, one-third discussed appropriate management protocols, and one-third exchanged information on necessary precautions. A majority (81%) consulted at least one other healthcare professional, most commonly the general practitioner (77.9%) and the oncologist (70.5%).

### Challenges faced by practitioners

While 20.9% of dental surgeons stated they experienced no difficulty in managing cancer patients, 79.1% reported specific challenges. The most frequently cited challenges were performing invasive procedures (46.7%), writing prescriptions (i.e. perceived complexity of safely prescribing pharmacological treatments) (45.8%), and completing medical questionnaires (i.e. dentists face challenges in gathering, interpreting, and documenting the complex and evolving medical histories of cancer patients to ensure safe dental care) (40.2%) (Table 1). Additionally, 38.2% reported experiencing at least two of these challenges, with 14% indicating three or more.

**Table 1:** Challenges reported by dental surgeons in managing cancer patients (N = 165)

Difficulty	Percentage (%)
No difficulties reported	20.9
Performing invasive procedures	46.7
Writing prescriptions	45.8
Completing medical questionnaires	40.2
Multiple difficulties (≥ 2)	38.2
Multiple difficulties (≥ 3)	14.0

Furthermore, 60% of respondents rated their difficulty in managing cancer patients as greater than 5 on a 10-point visual analog scale. The most challenging cancer types to treat were head and neck cancers (54%), followed by hematological malignancies (15%). Regarding treatments, dental surgeons reported the greatest challenges with radiotherapy (33%) and chemotherapy (27%).

### Knowledge of treatment toxicities

When asked to list cancer treatment toxicities, only 3.5% mentioned salivary changes and 3.5% mentioned mucositis, while 9.7% mentioned osteonecrosis. Regarding care adaptations, enhanced interprofessional communication was reported by 67%, modified appointment scheduling by 52%, and additional precautions during invasive procedures by 78%. Preventive measures such as oral hygiene instruction and fluoride application were mentioned by 7.8% of practitioners.

### Knowledge and training in supportive care

Of the 165 study participants, only 29% reported having knowledge of supportive care in oncology. Among those with knowledge, 46% acquired it during their initial training, 37% through continuing education, and 15% through both. For those who engaged in continuing education, 70% attended conferences, while private training (30%), university courses (26%), and online courses (15%) were less frequently utilized (Table 2).

**Table 2:** Training sources and needs in supportive care (N = 165)

Category	Percentage (%)
**Knowledge of Supportive Care**	
Reports having knowledge	29.0
Self-rated knowledge < 5/10	73.0
**Source of Knowledge (among those with knowledge)**	
Initial training	46.0
Continuing education	37.0
Both	15.0
**Continuing Education Format (among those with CE)**	
Conferences	70.0
Private training	30.0
University courses	26.0
Online courses	15.0
**Training Needs**	
Expressed need for further training	89.0
Current training opportunities insufficient	89.0

While 89% of responding dental surgeons felt concerned about supportive care in oncology, 73% rated their knowledge in this area as less than 5 out of 10, and 89% expressed a need for further training. Respondents indicated a strong preference for in-person workshops, online courses, and conferences as preferred training methods. A significant proportion also expressed a need for additional reading materials. Despite the evident need, 89% of respondents reported that current training opportunities in this field are insufficient.

## Discussion

Our study reveals that nearly all dental surgeons (96%) manage cancer patients, and a vast majority (89%) express concern about supportive care in oncology. These results highlight the profession’s collective awareness of the oral impact of cancer and the role dental surgeons can play in improving patients’ quality of life [11].

### Engagement with existing literature: contextualizing our findings

#### Cancer types and management challenges

Head and neck cancer treatments induce orofacial toxicities more frequently and severely than treatments for other cancer sites [5]. As expected, dental surgeons reported greater challenges in managing patients with these types of cancer. However, it is important to recognize that treatments for other cancer types can also have systemic effects impacting the oral cavity and quality of life [12].

Hematological malignancies ranked second in terms of challenging cancer types. Hematologic malignancies and their treatments (including bone marrow transplantation and immunosuppressants) cause severe immunodeficiency and blood cell alterations that directly impact oral health [13]. Dentists find these cancers challenging because any dental procedure carries high infection risks, requiring careful coordination with oncology teams to ensure patient safety.

#### Knowledge of treatment toxicities

Our study reveals concerning gaps in dental practitioners’ understanding of cancer treatment-related oral toxicities. While a substantial proportion of dental surgeons reported awareness of anticancer treatment toxicities, analysis of their specific knowledge demonstrates significant deficits that may impact patient care quality.

The low recognition rate of salivary dysfunction is particularly concerning given that xerostomia is consistently ranked among the most debilitating and persistent side effects experienced by cancer patients [14, 15]. This knowledge gap may compromise practitioners’ ability to anticipate and manage a condition that significantly impacts quality of life, nutrition, and oral health maintenance throughout survivorship.

Similarly, the limited recognition of mucositis represents a critical educational deficit. Oral mucositis is one of the most common and severe acute toxicities of cancer therapy, occurring in up to 90% of patients receiving high-dose chemotherapy or head and neck radiotherapy [16]. This suggests that many dental practitioners may be inadequately prepared to prevent or manage this debilitating condition during active treatment phases.

Conversely, the higher recognition of osteonecrosis, despite its relatively low spontaneous occurrence (0–1.9% depending on treatment) compared to xerostomia and mucositis [17], indicates a selective focus on complications with direct procedural implications [18, 19]. This pattern suggests that practitioners’ knowledge may be influenced more by immediate clinical concerns requiring treatment modifications rather than comprehensive understanding of patient-centered outcomes.

These findings indicate that current dental education may inadequately prepare practitioners to recognize and address the full spectrum of oral health needs in cancer patients, potentially limiting opportunities for preventive care and optimal supportive care integration.

#### Adaptation of care

The majority (85%) of surveyed dentists reported modifying their approach for cancer patients. The most significant adaptations included enhanced interprofessional communication, modified appointment scheduling, and additional precautions during invasive procedures. Dentists treating patients undergoing radiation therapy implemented heightened precautionary measures, particularly focusing on preventing osteonecrosis through prophylactic extractions and less invasive subsequent interventions [20].

However, preventive measures such as oral hygiene instruction and fluoride application were mentioned by less than 8% of practitioners, despite their established importance in preventing complications [21].

#### Training in supportive care

Only 29% of dental practitioners claimed knowledge of supportive oncology care, with only half attributing this to their initial education. These findings align with international studies highlighting variability in knowledge and practices regarding oral healthcare for cancer patients [22–24].

Scientific societies’ recommendations emphasize the importance of pre-treatment oral examination, basic oral hygiene, infection prevention, and post-treatment monitoring [26]. Many existing guidelines require adaptation for practical implementation in general dental practice settings, highlighting the need for more accessible, practice-oriented guidance for general dentists [26].

#### Areas for improvement

Communication challenges were evidenced by only 60% of practitioners contacting dental professionals at oncology centers, and the reported difficulties with completing medical questionnaires (40.2%) and coordinating care.

Due to the exploratory nature of the study and methodological constraints, no internal validity analysis (Cronbach’s alpha, factor analysis) or test–retest reliability assessment was conducted. This limitation should be taken into consideration when interpreting the results. Response bias may exist as respondents may represent dentists with greater interest in cancer patient care, potentially over-representing engagement with supportive care compared to the broader dental community. Additionally, as our study was limited to the Occitanie region, generalization of results to the entire French territory requires caution, although this region presents geographical and demographic diversity representative of France. To address these issues, we propose:

- **Development of targeted training programs:** Both initial dental education and continuing education in France should incorporate more content on supportive care for cancer patients. These could range from short conferences to specialized courses or university diplomas, delivered in French and adapted to the French healthcare system. Targeted training will facilitate interdisciplinary collaboration with medical, nursing, and dietetic teams within French cancer centers, promoting improved oral health management for cancer patients.
- **Enhanced interprofessional communication:** Clear information pathways should be established between French oncology services and dental practitioners to ensure optimal patient care within the framework of the French healthcare system.

## Conclusion

By addressing perceived barriers to care, such as lack of communication and training, it is possible to improve the overall quality of life for cancer patients. This study reveals that while French dentists demonstrate concern about cancer patient care, significant knowledge gaps exist regarding supportive care. Despite 96% treating cancer patients, only 29% report knowledge of supportive care principles, and 89% express need for additional training. These findings highlight a disconnect between clinical involvement and educational preparedness in managing cancer patients’ oral health needs. To address these identified gaps, we recommend: (1) enhanced training programs in initial and continuing dental education specifically designed for French dental professionals; (2) improved communication pathways between oncology services and dental practitioners within the French healthcare system; and (3) implementation of translated, profession-specific guidelines for dental management of cancer patients adapted to the French healthcare context. Addressing these educational and communication barriers represents a critical step toward optimizing supportive care delivery and improving quality of life for cancer patients in France.

## Supplementary Information

Below is the link to the electronic supplementary material.

ESM1 (PDF 269 KB)
