# Supplementary material for: Dentists’ knowledge and practices in supportive care: a French national survey
Source: Support Care Cancer. 2026 Feb 23;34(3):235. doi: 10.1007/s00520-026-10392-9 (PMC12929310; doi:10.1007/s00520-026-10392-9)
Supplement: Supplementary file 1 — (PDF 269 KB) [file 520_2026_10392_MOESM1_ESM.pdf]

ANNEXES

Annexe 1 : Questionnaire

Le chirurgien-dentiste et les soins oncologiques de support.

Les soins de support désignent l'ensemble des soins et soutiens nécessaires aux personnes malades, parallèlement aux traitements spécifiques, lorsqu'il y en a, tout au long des maladies graves.

Voici un questionnaire adressé aux chirurgiens-dentistes de France.
Ce questionnaire a pour but de faire un état des lieux sur la prise en charge des patients atteints de cancer par les praticiens français et leur place dans les soins oncologiques de support, ainsi que sur la formation à ce sujet et les axes d'amélioration.

Concernant votre formation de chirurgien-dentiste

1. Votre département d'exercice ?

2. En quelle année avez-vous été diplômé ? (Année de thèse)

3. Lieu d'obtention du diplôme :

4. Êtes-vous interne ou ancien interne ?

   Une seule réponse possible.

   - Oui
   - Non

5. Si oui, dans quelle spécialité ?

   Une seule réponse possible.

   - Médecine Bucco-Dentaire
   - Orthopédie Dento-Faciale
   - Chirurgie Orale

6. Si oui, dans quelle ville avez-vous réalisé votre internat ?

Concernant votre prise en charge des patients atteints ou ayant eu un cancer

7. Dans votre patientèle, avez-vous des patients atteints de cancer ou en cours de prise en charge oncologique ?

Une seule réponse possible.

- ( ) Oui
- ( ) Non

Si oui, connaissez-vous :

8. La localisation ?

Plusieurs réponses possibles.

- [ ] ORL
- [ ] Poumon
- [ ] Sein
- [ ] Prostate
- [ ] Hémopathie maligne

Autre : [ ] ______________________

9. Le type des thérapeutiques anti-cancéreuses reçues ?

Plusieurs réponses possibles.

- [ ] Chirurgie
- [ ] Radiothérapie
- [ ] Chimiothérapie
- [ ] Thérapie ciblée
- [ ] Immunothérapie
- [ ] Hormonothérapie

Autre : [ ] ______________________

10. Les risques en fonction de la thérapie anticancéreuse à prendre en compte pour ces patients ?

Une seule réponse possible.

- ( ) Oui
- ( ) Non

11. Si oui, lesquels ?

____

12. Les toxicités potentielles des traitements anticancéreux à court et long terme ?

Une seule réponse possible.

- ( ) Oui
- ( ) Non

13. Si oui, lesquelles ?

____

14. Modifiez-vous votre approche et prenez-vous des précautions pour ces patients ?

Une seule réponse possible.

- ( ) Oui
- ( ) Non

15. Si oui, de quelle façon ? (Organisationnelle, plateau technique...)

____

16. Avez-vous contacté le praticien de médecine bucco-dentaire du centre anti-cancéreux ou l'équipe oncologique :

Plusieurs réponses possibles.

- [ ] afin d'avoir des renseignements sur les traitements reçus
- [ ] pour échanger sur la prise en charge
- [ ] pour vous renseigner sur les précautions à prendre
- [ ] non

Autre : [ ] ____________________

17. Si non, pourquoi ?

Plusieurs réponses possibles.

- [ ] Suite à des difficultés pour contacter l'équipe médicale.
- [ ] Vous n'en avez pas eu la nécessité

Autre : [ ] ____________________

18. Avez-vous contacté d'autres intervenants de santé pour coordonner leur prise en charge ?

Plusieurs réponses possibles.

- [ ] Oncologue
- [ ] Radiothérapeute
- [ ] Chirurgien
- [ ] Médecin traitant
- [ ] Kinésithérapeute
- [ ] Orthophoniste
- [ ] Diététicien
- [ ] Psychologue
- [ ] Aucun

Autre : [ ] ____________________

Difficultés ressenties dans la prise en charge de patients atteints de cancer

19. Dans la prise en charge de ces patients, avez-vous ressenti des difficultés :

Plusieurs réponses possibles.

- [ ] dans le dialogue avec le patient
- [ ] lors du questionnaire médical
- [ ] lors de l'examen clinique
- [ ] lors des soins invasifs
- [ ] lors de la rédaction de l'ordonnance
- [ ] Aucune difficulté

Autre : [ ] ____________________

20. Veuillez développer les difficultés rencontrées :

____

21. Sur une échelle de 0 à 10 : évaluez votre difficulté globale dans la prise en charge de ces patients

Une seule réponse possible.

	1	2	3	4	5	6	7	8	9	10	
Aucune difficulté	○	○	○	○	○	○	○	○	○	○	Grande difficulté

22. Avez-vous rencontré plus de difficultés dans la prise en charge de certaines localisations par rapport à d'autres ?

Plusieurs réponses possibles.

- [ ] ORL
- [ ] Poumon
- [ ] Sein
- [ ] Prostate
- [ ] Hémopathie maligne
- [ ] Aucune différence

Autre : [ ] ______________________

23. Avez-vous rencontré plus de difficultés en fonction des thérapeutiques anti cancéreuses reçues ?

Plusieurs réponses possibles.

- [ ] Chirurgie
- [ ] Radithérapie
- [ ] Chimiothérapie
- [ ] Thérapie ciblée
- [ ] Immunothérapie
- [ ] Hormonothérapie
- [ ] Aucune différence

Autre : [ ] ______________________

Soins oncologiques de support (SOS) et accès aux informations

24. Avez-vous des connaissances en soins oncologiques de support ?

Une seule réponse possible.

( ) Oui

( ) Non

25. Si oui, à quel moment avez-vous acquis ces connaissances ?

Plusieurs réponses possibles.

- [ ] Formation initiale
- [ ] Formation continue

Autre : [ ] ______________________

26. Dans le cas de la formation continue, sous quel format ?

Plusieurs réponses possibles.

- [ ] Formation Universitaire (DU, DIU, AEU, ...)
- [ ] Formation privée
- [ ] Conférence
- [ ] Formation en ligne

Autre : [ ] ______________________

27. En tant que chirurgien-dentiste, vous sentez-vous concerné par les soins oncologiques de support ?

Une seule réponse possible.

( ) Oui
( ) Non

28. Sur une échelle de 0 à 10, comment évalueriez-vous vos connaissances pour la prise en charge de ces patients ?

Une seule réponse possible.

	1	2	3	4	5	6	7	8	9	10	
Aucune connaissance	( )	( )	( )	( )	( )	( )	( )	( )	( )	( )	Prise en charge optimale

29. Ressentez-vous le besoin d'approfondir vos connaissances dans ce domaine ?

Une seule réponse possible.

( ) Oui
( ) Non

30. Si oui, sous quel format ?

____

31. Comment évalueriez-vous l'offre de formations à ce sujet ?

Une seule réponse possible.

	1	2	3	4	5	6	7	8	9	10
Inexistante	( )	( )	( )	( )	( )	( )	( )	( )	( )	( )
